# Self-Harm and Suicide Attempts among High-Risk, Urban Youth in the U.S.: Shared and Unique Risk and Protective Factors

**DOI:** 10.3390/ijerph9010178

**Published:** 2012-01-11

**Authors:** Monica H. Swahn, Bina Ali, Robert M. Bossarte, Manfred Van Dulmen, Alex Crosby, Angela C. Jones, Katherine C. Schinka

**Affiliations:** 1 Institute of Public Health, Georgia State University, 50 Decatur Street Southeast, Atlanta, GA 30303, USA; Email: bina226@gmail.com; 2 Department of Psychiatry, University of Rochester, 400 Fort Hill Avenue, Canandaigua, Rochester, NY 14424, USA; Email: robert_bossarte@urmc.rochester.edu; 3 Department of Psychology, Kent State University, 800 East Summit Street, Kent, OH 44242, USA; Email: manfredathome@gmail.com (M.V.D.); kschinka@gmail.com (K.C.S.); 4 Division of Violence Prevention, National Center for Injury Prevention and Control, Centers for Disease Control and Prevention, 1600 Clifton Road Northeast, Atlanta, GA 30329, USA; Email: aec1@cdc.gov; 5 Department of Psychology, John Carroll University, 20700 North Park Boulevard, University Height, OH 44118, USA; Email: acjones@jcu.edu

**Keywords:** self-harm, suicide attempt, youth, adolescents, U.S., high-risk, school, cross-sectional

## Abstract

The extent to which self-harm and suicidal behavior overlap in community samples of vulnerable youth is not well known. Secondary analyses were conducted of the “linkages study” (N = 4,131), a cross-sectional survey of students enrolled in grades 7, 9, 11/12 in a high-risk community in the U.S. in 2004. Analyses were conducted to determine the risk and protective factors (*i.e.*, academic grades, binge drinking, illicit drug use, weapon carrying, child maltreatment, social support, depression, impulsivity, self-efficacy, parental support, and parental monitoring) associated with both self-harm and suicide attempt. Findings show that 7.5% of participants reported both self-harm and suicide attempt, 2.2% of participants reported suicide attempt only, and 12.4% of participants reported self-harm only. Shared risk factors for co-occurring self-harm and suicide attempt include depression, binge drinking, weapon carrying, child maltreatment, and impulsivity. There were also important differences by sex, grade level, and race/ethnicity that should be considered for future research. The findings show that there is significant overlap in the modifiable risk factors associated with self-harm and suicide attempt that can be targeted for future research and prevention strategies.

## 1. Introduction

Self-harm is a prevalent and complex problem that primarily affects adolescents and young adults [1,2,3,4,5]. In 2007, there were an estimated 77,024 injuries treated in U.S. emergency departments for self-harm that involved cutting or piercing [6], the most common forms of self-harm [7]. However, most youth who harm themselves in the U.S. do not seek treatment [8]. Suicidal behaviors, that is self harm with the intent to die, is less prevalent than self-harm without the intent to die [9]; however, the behaviors are complex and interrelated [10]. An estimated 70% of adolescents who engage in repetitive self-harm also attempt suicide [11]. In one survey 9.7% of adolescents reported having ever attempted suicide [12] and the estimated lifetime prevalence of self-harm ranges between 13.0% and 23.2% [3], although a higher prevalence (46.5%) has been reported [13]. A recent study of middle school students found that 28.4% reported self-harm in the past year [14]. 

Despite an increased awareness and research of self-injurious behaviors among adolescents [3], epidemiological studies of the risk factors of self-harm remain relatively scarce [13,14,15]. Even less is known about potentially shared or unique factors associated with self-harm and suicidal behaviors. While self-harm is a major risk factor for suicide [16,17,18,19], the extent to which self-harm and suicidal behavior overlap in community samples of vulnerable youth is less known. Moreover, few studies have examined the risk factors that contribute to self-harm, while considering the impact of suicidal history [20,21]. This is an important area for research because self-harm is typically a means of expressing self-directed anger or resisting suicidal thoughts [22] suggesting possible separate etiologies. Self-harm is also used to signal distress and distract from painful feelings when other communication strategies have failed [23]. Furthermore, empirical research findings indicate that those who engage in self-harming often have psychological problems including depression, anxiety, impulsivity, low self-esteem and suicidal ideation [2,7,8,21,24,25,26,27].

Previous studies show that low socio-economic status, behavior problems, somatic problems, eating disorders, thought problems, poor emotion regulation, poor communication, child maltreatment, delinquent and aggressive behaviors and substance use are associated with adolescent’s suicide ideation or self-harm behavior [28,29,30,31,32,33,34,35,36]. Although studies have examined the association between self-harm and suicide [16,17,18,19], there is limited information about the potentially shared and unique risk factors for self-harm and suicide attempts. The research question guiding the current study is whether or not suicidal behavior and self-harming have shared or unique risk and protective factors. Findings from this study will add to the relatively limited information currently available about co-occurring self-harm and suicidal behaviors among urban youth. 

## 2. Method

The “Youth Violence Survey: Linkages among Different forms of Violence” was administered to all public school students enrolled in grades 7, 9, 11 and 12 in a school district in a high-risk community in the U.S in 2004. The details of the study have been described elsewhere [36,37,38]. The school district was identified and selected using community indicators of risk (*i.e.*, poverty, unemployment, single parent households, and serious crimes), it was racially and ethnically diverse, and it was located in a city with a population of less than 250,000. This district operated 16 schools (elementary, middle, high schools, alternative schools) which all agreed to participate in the study. Within these 16 schools, all students in grades 7, 9, 11, and 12 were invited to participate. Students in grades 11 and 12 were grouped to produce a sufficient number of participants in the oldest of the three age groups.

### 2.1. Procedures

Data collection occurred in April 2004. Students voluntarily completed the anonymous, self-administered 174-item questionnaire in classrooms during a 40-minute class period. Students without parental permission or who did not want to participate in the study were assigned individual deskwork (by the classroom teacher), which they completed at their desks or at an alternate location designated by the school during the survey administration. The questionnaire, an optically scannable booklet in multiple-choice format, was administered by highly experienced field staff. All English-speaking students in the targeted grades were invited to participate in the study. However, students who could not complete the questionnaire independently (e.g., enrolled in a special education class, required the assistance of a translator, had cognitive disabilities that would prevent adequate understanding and responding to the survey; n = 151), or who were no longer attending school (e.g., had dropped out of school, had been expelled, or were on long-term out-of-school suspension; n = 202), were ineligible to participate in the study.

Prior to data collection, active, signed, written parental permission, and student assent were required for all students under 18 years of age to participate in the study. Students aged 18 years or older provided written consent prior to participating in the survey. Parental permission forms were provided in English, Spanish, and other major languages as requested by the schools. Students received a $5 gift card for returning the parental permission form regardless of whether the parent approved or denied the student’s participation in the survey. Students who completed the survey received an additional $5 gift card. Return of the parental permission form by invited students was high (86% of students returned the form), and parent and student refusals were very low (approximately 1% each). Of the 5,098 students who met eligibility criteria, 4,131 participated, yielding a participation rate of 81%: 1,491 in 7th grade (83.0%), 1,117 in 9th grade (73.4%), and 1,523 in 11th and 12th grades combined (79.0%). The study received IRB approval from the Centers for Disease Control and Prevention and ORC Macro International. IRB approval was also obtained at Georgia State University for continuation of secondary analyses of these data.

### 2.2. Measures

Self-harm was assessed through a single item reflecting the number of times the student had deliberately harmed or injured themselves in the past year, even if they did not intend to die. Suicide attempt was also assessed through a single item reflecting whether the student had attempted suicide at least once in the past year. Responses to both measures were dichotomized. 

Other risk factors assessed included academic grades, binge drinking, illicit drug use, weapon carrying, child maltreatment, social support, depression, impulsivity, self-efficacy, parental support, and parental monitoring. These measures have been described elsewhere [36,37,38,39,40,41,42,43]. 

Briefly, most measures were dichotomized as follows: academic performance (having A’s and B’s during the past 12 months); binge drinking (five or more drinks at any time in past year); any illicit drug use (any use of inhalants/illegal drugs); weapon carrying (gun, knife or club, past month); and any child maltreatment (exposure to domestic violence, physical, or sexual victimization prior to age 10). A few continuous measures were included such as social support, a 9-item measure (α = 0.76), based on the Vaux Social Support Scale [40,44,45] assessing peer, family and school contexts. Depression, a 6-item modified measure (α = 0.85), assessed how many times participants had been sad, grouchy or irritable or moody, hopeless, not eating, changes in sleep and difficulty concentrating in the past month [46]. Impulsivity, a 4-item measure (α = 0.79), asked if participants had a hard time sitting, finishing things, did things without thinking, and needed to use a lot of self-control to keep out of trouble [47]. Self-efficacy to avoid violence [48], a 7-item scale (α = 0.88), assessed participants’ confidence for staying out of fights and resolving conflict. Parental support, a 5-item scale (α = 0.79), assessed parents/guardians’ reinforcement for positive behaviors in the past month and parental monitoring, a 4-item scale (α = 0.76), assessed parents/guardians’ child-monitoring strategies in the past month [48]. 

### 2.3. Analysis

Measures of self-harm and suicide attempts were combined into four mutually exclusive groups: (1) self harm only, (2) suicide attempts only, (3) both self-harm and suicide attempts, and (4) neither. Chi-square analyses and multilogistic regression analyses were used to test associations of self-harm and suicide attempt with demographic characteristics and risk factors. T-tests were used to determine mean differences in social support, depression, impulsivity, self-efficacy, parental support, and parental monitoring for those reporting self harm or suicide attempts.

## 3. Results

The prevalence of self-harm (in the past year) was 20.3% (23.9% for girls and 16.4% for boys). Moreover, among those who reported self-harm, 38.2% had also attempted suicide in the past year). Findings based on the four-level outcome variable show that 7.5% of participants reported both self-harm and suicide attempt, 2.2% of participants reported suicide attempt only, and 12.4% of participants reported self-harm only. Findings from the chi-square analyses and t-tests show that all independent variables examined were significantly associated with self-harm and suicide attempt (Table 1). Moreover, patterns of the outcome variables varied significantly by sex, grade level and race/ethnicity (Table 1). More specifically, girls, and students with Hispanic ethnicity were most likely to report both suicide attempt and self-harm in the past year. Additionally, students who reported binge drinking, illicit drug use, weapon carrying child maltreatment were also more likely to report both suicide attempt and self-harm. Similarly, students who reported less social support, higher levels of depression, higher levels of impulsivity, lower levels of self-efficacy, lower levels of parental support and lower levels of parental monitoring were also more likely to report both suicide attempt and self-harm.

The multivariate analyses showed that child maltreatment (Adjusted OR = 2.13; 95% CI: 1.50, 3.01), weapon carrying (Adjusted OR = 3.31; 95% CI: 2.25, 4.86), binge drinking (Adjusted OR = 1.75; 95% CI: 1.21, 2.54), depression (Adjusted OR = 3.31; 95% CI: 2.71, 4.03), and impulsivity (Adjusted OR = 1.44; 95% CI: 1.22, 1.69) were significantly associated with co-occurring self-harm and suicide relative to those who reported neither (Table 2). Similarly, child maltreatment (Adjusted OR = 1.56; 95% CI: 1.24, 1.97), depression (Adjusted OR = 2.14; 95% CI: 1.85, 2.46), and impulsivity (Adjusted OR = 1.25; 95% CI: 1.10, 1.41) were associated with self-harm only. Weapon carrying (Adjusted OR = 2.16; 95% CI: 1.10, 4.23), binge drinking (Adjusted OR = 2.04; 95% CI: 1.15, 3.61), illicit drug use (Adjusted OR = 1.81; 95% CI: 1.07, 3.05), and depression (Adjusted OR = 2.52; 95% CI: 1.86, 3.40) were associated with suicide attempt only. Parental support (Adjusted OR = 0.50; 95% CI: 0.33, 0.76) was associated with a decreased likelihood of reporting co-occurring self-harm and suicide. 

## 4. Discussion

This cross-sectional study of youth in an urban area examined the prevalence and psychosocial correlates associated with reporting both self-harm and suicide attempts. The findings show that the demographic and psychosocial factors associated with both suicide attempt and self-harm vary significantly. Girls and students in the younger grade levels were most likely to report both self-harm and suicide attempts. Moreover the findings show that there is a significant overlap in the modifiable risk factors associated with self-harm and suicide attempt that can be targeted for future research and prevention strategies. More specifically, binge drinking, weapon carrying, child maltreatment, depression, impulsivity and lower levels of parental support were specifically associated with both self-harm and suicide attempt.

With respect to the demographic characteristics associated with both self-harm and suicide attempt, it is clear that girls are at the highest risk, as are those youth who engage in or are exposed to other high-risk behaviors and past victimization. This is not surprising given previous research that show that being female, low socio-economic status, behavior problems, somatic problems, eating disorders, thought problems, delinquent behavior, substance use, and aggressive behavior are associated with adolescent’s suicide ideation or self-harm behavior. [28,34,35,39,49,50]. 

**Table 1:** Demographic and psychosocial characteristics associated with self-harm or suicide attempts among high-risk urban youth in the U.S.

	Both Self-Harm and Suicide Attempt		Suicide Attempt Only		Self-Harm Only		Neither Self-Harm Nor Suicide Attempt		
	N	%/Mean (SD)	N	%/Mean (SD)	N	%/Mean (SD)	N	%/Mean (SD)	*p*-value *
Sex									
Girls	210	10.24	57	2.78	271	13.21	1,513	73.77	<0.0001
Boys	85	4.50	28	1.48	219	11.59	1,557	82.42	
Race/ethnicity									
Hispanics	156	9.03	38	2.20	210	12.16	1,323	76.61	<0.0001
African Americans	52	4.85	33	3.08	95	8.86	892	83.21	
Whites	72	8.20	8	0.91	144	16.40	654	74.49	
Others	14	7.49	1	0.53	32	17.11	140	74.87	
Grade									
7th	121	8.77	27	1.96	165	11.96	1,067	77.32	<0.05
9th	94	8.73	20	1.86	131	12.16	832	77.25	
11/12th	81	5.45	37	2.49	195	13.13	1,172	78.92	
Academic grades									
Mostly A’s and B’s	170	8.60	41	2.07	252	12.75	1,514	76.58	<0.05
Mostly C’s, D’s and F’s	108	6.13	33	1.87	220	12.49	1,400	79.50	
Binge drinking									
No	169	5.68	51	1.71	331	11.12	2,425	81.49	<0.0001
Yes	129	13.12	34	3.46	161	16.38	659	67.04	
Illicit drug use									
No	172	5.78	52	1.75	327	10.98	2,427	81.50	<0.0001
Yes	118	12.95	33	3.62	156	17.12	604	66.30	
	**Both Self-Harm and Suicide Attempt**		**Suicide Attempt Only**		**Self-Harm Only**		**Neither Self-Harm Nor Suicide Attempt**		
	**N**	**%/Mean (SD)**	**N**	**%/Mean (SD)**	**N**	**%/Mean (SD)**	**N**	**%/Mean (SD)**	*p*-value */ ***F*-value**
Weapon carrying									
No	182	5.57	62	1.90	383	11.72	2,640	80.81	*<0.0001*
Yes	110	16.72	23	3.50	106	16.11	419	63.68	
Child maltreatment									
No	70	3.15	36	1.62	200	8.99	1,918	86.24	*<0.0001*
Yes	226	13.08	49	2.84	291	16.84	1,162	67.25	
Mean social support	291	2.07 (0.48)	84	2.19 (0.49)	486	2.17 (0.44)	3,007	2.22 (0.50)	*<0.0001*
Mean depression	294	3.64 (0.90)	84	3.20 (0.91)	488	3.12 (0.89)	3,054	2.42 (0.87)	*<0.0001*
Mean impulsivity	289	3.15 (1.05)	83	2.63 (1.05)	480	2.77 (0.98)	2,999	2.25 (0.95)	*<0.0001*
Mean efficacy	296	2.99 (1.04)	85	3.17 (1.15)	487	3.18 (1.01)	3,040	3.35 (1.08)	*<0.0001*
Parental support	298	1.91 (0.51)	85	2.07 (0.48)	491	2.06 (0.48)	3,072	2.19 (0.48)	*<0.0001*
Parental monitoring	298	2.14 (0.50)	85	2.19 (0.50)	492	2.21 (0.46)	3,076	2.29 (0.47)	*<0.0001*

* Wald Chi-Square test of the association between demographic and psychosocial factors and self-harm for dichotomous variables and t-test for differences in means for continuous variables.

**Table 2:** Multivariate associations between demographic and psychosocial characteristics and self-harm and suicide attempts among high-risk urban youth in the U.S.

	Both Self-Harm and Suicide Attempt	Suicide Attempt Only	Self-Harm Only
	Adj. OR (95% CI)	Adj. OR (95% CI)	Adj. OR (95% CI)
Girls	**2.28 (1.58, 3.29)**	1.57 (0.88, 2.83)	0.94 (0.73, 1.19)
Race/ethnicity			
Hispanics	0.82 (0.56, 1.18)	**3.82 (1.31, 11.17)**	**0.67 (0.51, 0.88)**
African Americans	**0.56 (0.36, 0.89)**	**5.19 (1.83, 14.77)**	**0.51 (0.37, 0.70)**
Others	0.65 (0.32, 1.33)	1.02 (0.11, 9.20)	0.88 (0.54, 1.44)
Grade			
9th	**0.61 (0.42, 0.89)**	0.77 (0.39, 1.52)	0.81 (0.61, 1.08)
11/12th	**0.29 (0.19, 0.45)**	0.55 (0.28, 1.07)	**0.71 (0.54, 0.95)**
High academic grades	0.80 (0.57, 1.11)	0.76 (0.45, 1.29)	0.83 (0.66, 1.05)
Binge drinking	**1.75 (1.21, 2.54)**	**2.04 (1.15, 3.61)**	1.10 (0.83, 1.46)
Illicit drug use	1.30 (0.90, 1.86)	**1.81 (1.07, 3.05)**	1.32 (1.00, 1.74)
Weapon carrying	**3.31 (2.25, 4.86)**	**2.16 (1.10, 4.23)**	1.35 (0.99, 1.84)
Child maltreatment	**2.13 (1.50, 3.01)**	0.87 (0.51, 1.50)	**1.56 (1.24, 1.97)**
Social support ^1^	1.09 (0.72, 1.63)	1.50 (0.76, 2.97)	1.19 (0.88, 1.59)
Depression ^1^	**3.31 (2.71, 4.03)**	**2.52 (1.86, 3.40)**	**2.14 (1.85, 2.46)**
Impulsivity ^1^	**1.44 (1.22, 1.69)**	1.04 (0.78, 1.39)	**1.25 (1.10, 1.41)**
Efficacy ^1^	1.09 (0.93, 1.27)	1.04 (0.76, 1.43)	0.99 (0.88, 1.12)
Parental support ^1^	**0.50 (0.33, 0.76)**	0.69 (0.38, 1.27)	0.75 (0.56, 1.01)
Parental monitoring ^1^	1.08 (0.75, 1.54)	0.86 (0.47, 1.56)	1.03 (0.79, 1.34)

^1^ Continuous variable; AOR = Adjusted Odds Ratio; Reference categories are boys, Whites, 7th grade, who had low academic grades (grades other than A’s and B’s), did not binge drink, did not use illicit drugs, did not carry weapon, did not experience child maltreatment, had social support, did not have depression, did not have impulsivity, had efficacy, had parental support, and had parental monitoring.

While sadness was highly associated with both self-harm and suicide attempt, suicide attempt only and self-harm only, the students who reported both self-harm and suicide attempt had higher prevalence of child maltreatment, impulsivity and less parental support when compared to students who reported suicide attempt only, self-harm only. This is an intriguing finding and suggests that there may be a potentially unique pattern of risk among youth who express both self-harm and suicide attempt. Previous research has established a strong association between non-suicidal self-harm behaviors and suicidal behaviors that also show that self-harm is a major risk factor for suicide or suicidal behaviors [3,14,16,17,18,19,39]. Child maltreatment, in particular sexual abuse, has been reported as an important predictor of self-harm [2,3] which is also a strong finding of our study. However, our study extends the previous literature by documenting that in this population these factors are uniquely associated with reporting both self-harm and suicide attempt and not with suicide attempt without self-harm. 

To place these findings in context, it may also be important to discuss the past year prevalence of self-harm among students in this study which was was relatively high, 20% overall (24% for girls and 16% for boys). Most other studies have reported a life time prevalence of self-harm within the range of 13% and 23% [3]. One recent study reported a past year prevalence of 28% among middle school students [14]. However, these varying ranges reflect different age intervals used for participants as well as somewhat different definitions of self-harm across studies. However, in general, our participants had a relatively high level of self-harm.

It is also within this context that our study found a substantial overlap between reporting self-harm and also reporting suicide attempts. In our study, among those who reported self-harm, 38.2% had also attempted suicide in the past year. Researchers have sought to differentiate between suicidal and non-suicidal self-harm [26]. However, the overlap of self-reported self-harm and suicidal behaviors indicated among participants in our study, raises more questions about the “comorbidity” of these behaviors as well as the underlying health issues, past experiences, and limited coping strategies including impulsivity. These findings are also corroborated by previous research documenting associations between self-harm behavior, poor emotion regulation, and poor communication skills [30,32]. These deficits may in turn exacerbate prior violent experiences and further increase the psychosocial disadvantage experienced by these youth. Our study also found that youth who had parental support was less likely to report both self-harm and suicidal behaviors. As such, increasing social support for these youth is an important priority. 

There are several noteworthy limitations that should be considered when interpreting these findings. First, measures were self-reported and may reflect biases, especially underreporting of sensitive information. Second, the data represent students in a high-risk urban community and may not generalize to adolescents in other communities or countries or those who have dropped out of school. The U.S. community where the study was conducted was particularly resource poor and may be limited in terms of available mental health services and general care. Third, analyses are based on cross-sectional data, which do not allow temporal ordering between correlates and outcomes. Accordingly, the analyses simply address the extent to which psychosocial correlates were associated with the outcome variable of interest. Fourth, the study did not assess other potential confounders or mediators that may have been important in the associations examined. Several important factors such as the use of coping strategies, cognitive functioning and communication strategies in addition to past help-seeking were not available within the existing dataset but could have been helpful in the explanation of factors associated with self-harm and suicide attempt. Finally, many of the measures included in the analyses were based on single items rather than comprehensive scales. 

Despite these limitations, the findings from this study can be used to empirically document a relatively high overlap between self-harm and suicidal behaviors among youth in a disadvantaged urban community in the U.S. Moreover, it appears that there are shared and unique factors that may be incorporated in future research and prevention programs of self-harm and suicidal behaviors among vulnerable youth across settings [51,52]. Unfortunately, public health prevention strategies that specifically address self-harm in youth populations is not available in the U.S. However, there are several different types of programs that may have some success in addressing self-harming behavior either by directly addressing self-harm or indirectly by addressing the associated risk factors such as prior victimization [39]. For example, the Guidelines for school response in the U.S. [53] and family involvement through collaborative strengths-based brief therapy model [54] may benefit self-harming adolescents [39]. Moreover, clinical treatments, while not feasible from a public health prevention perspective, show some efficacy, specifically the cognitive behavioral therapies (CBT) [55]. 

Despite the increased interest and pursuit of research into self-harm, its etiology and epidemiology, many aspects of self-harm remain relatively poorly understood. As such, future research is clearly needed to better understand and respond to the growing need of youth who experience self-harm and suicidal behaviors. Previous priorities have been outlined for this field of research [3]. However, one of the key barriers to progress in this field is the scarcity of available data sources that have included measures of non-suicidal self-injury or self-harm. A recommendation for future data collections is to incorporate measures of self-harm, particularly among adolescents and young adults, so that the prevalence and epidemiology of self-harm can be studied across a range of populations and settings. Finally, while self-harm is clearly a complex and multi-faceted problem [1], efforts that seek to understand this issue better and that find ways to develop prevention and intervention strategies are sorely needed. 
